# Fluorescent Imprinted Nanoparticles for Sensing of Chlorogenic Acid in Coffee Extracts

**DOI:** 10.3390/s22249874

**Published:** 2022-12-15

**Authors:** Anggy Lusanna Gutiérrez-Ortiz, Veronica Vida, Matjaž Peterka, Jasmina Tušar, Federico Berti, Luciano Navarini, Cristina Forzato

**Affiliations:** 1 Dipartimento di Scienze Chimiche e Farmaceutiche, Università degli Studi di Trieste, Via Giorgieri 1, 34127 Trieste, Italy; 2 COBIK, Mirce 21, 5270 Ajdovščina, Slovenia; 3 Illycaffè S.p.A., Via Flavia 110, 34147 Trieste, Italy

**Keywords:** polyphenols, chlorogenic acids, molecularly imprinted polymers, fluorescence, sensor

## Abstract

Green coffee beans are particularly rich in chlorogenic acids (CGAs), and their identification and quantification are usually performed by HPLC, coupled with mass spectrometry (LC-MS). Although there are a few examples of molecularly imprinted polymers (MIPs) for chlorogenic acid (5-CQA) recognition present in the literature, none of them are based on optical fluorescence, which is very interesting given its great sensitivity. In the present manuscript, fluorescent polymeric imprinted nanoparticles were synthetized following the non-covalent approach using hydrogenated 5-*O*-caffeoylquinic acid (H-5-CQA) as the template. The capability of the polymer to bind 5-CQA was evaluated by HPLC and fluorescence. A real sample of coffee extract was also analyzed to verify the selectivity of the polymer. Polymer fMIP01, containing 4-vinylpyridine and a naphtalimide derivative as monomers, showed a good response to the fluorescence quenching in the range 39 μM–80 mM. In the real sample, fMIP01 was able to selectively bind 5-CQA, while caffeine was not recognized. To demonstrate this, there is a promising system that can be exploited in the design of an optical sensor for 5-CQA detection. Polymer fMIP01 was immobilized by physical entrapment on a functionalized glass surface, showing a quenching of fluorescence with an increase of the CGA concentration between 156 μM and 40 mM.

## 1. Introduction

**Figure 1:**
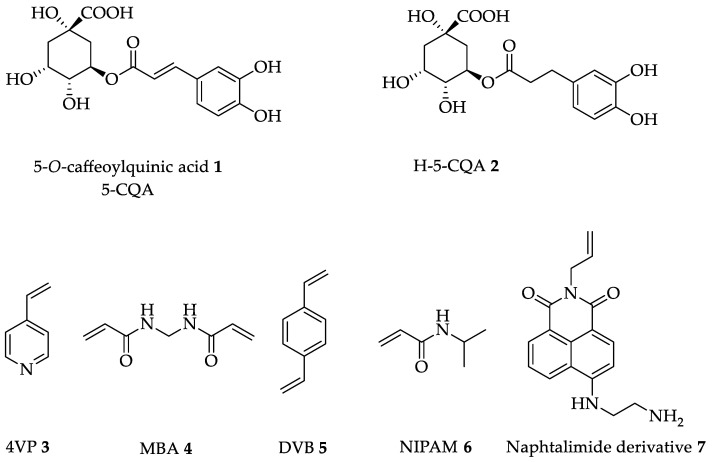
Chemical structures of analyte **1**, template **2**, monomers **3** and **7**, co-monomer **6** and cross-linkers **4** and **5**.

Chlorogenic acids (CGAs) are polyphenols widely present in the plant kingdom, and they are secondary metabolites involved in the defense mechanism against environmental stress of plants. Green coffee beans are particularly rich in these compounds, which are considered as indicators of the quality of coffee, since they affect the flavor and the nutritional value of the final product. Although the highest concentration of CGAs is in coffee, they can also be found in tea, berry fruits, cocoa, citrus fruits, apples, and pears [1]. CGAs possess several biological activities, such as being antioxidants, antibacterials, hepatoprotectives, cardioprotectives, anti-inflammatory agents, antipyretics, neuroprotectives, anti-obesity agents, antivirals, anti-microbials, anti-hypertension agents, free radical scavengers, and central nervous system (CNS) stimulators, providing health benefits upon consumption [2]. Chemically, CGAs are a class of esters of (-)-quinic acid with different cinnamic acids, and due to the different hydroxyl groups present in the quinic acid structure and the different cinnamic acids present in nature (caffeic acid, ferulic acid, *p*-coumaric acid, and sinapic acid), it is possible to form different monoesters, as well as diesters or triesters. A total number of 76 different CGAs has been recognized in the last years in coffee, but the most abundant is 5-*O*-caffeoylquinic acid (5-CQA) **1**, which is simply named chlorogenic acid. (Figure 1) The total content of CGAs in coffee depends on the coffee species (*Coffea arabica* 4–8% and *Coffea canephora* 7–14% on the dry matter basis), but also on the degree of roasting, the agricultural practices, as well as the soil composition.

The identification and quantification of CGAs in coffee is usually performed by HPLC coupled with mass spectrometry (LC-MS), and the different types of CGAs are recognized by the different fragmentation patterns according to their corresponding base ions [3]. In the literature, the characterization of CGAs was achieved by LC-ion trap-MSn, using fragment-targeted MSn, without the need of isolation of the products. Other analytical methods have been used, such as infrared spectroscopy, nuclear magnetic resonance (NMR), ultraviolet spectroscopy (UV-Vis), or chemiluminescence, but these methods are expensive and time consuming.

In recent years, attention has been paid to the determination of CGAs by molecularly imprinted polymers (MIP), which offer the possibility of specific molecular recognition. MIPs are synthetic materials able to specifically and selectively recognize a target compound, and their preparation starts from the formation of a complex between a target molecule, that acts as a template, and one or more functional monomers, in which are present acryloyl- or vinyl- groups that allow polymerization and which are chosen based on their ability of interacting with the functional groups of the template (analyte). The synthesis is carried out in the presence of a cross-linking agent and a radical initiator in a suitable porogenic solvent, leading to the formation of a rigid three-dimensional network around the template. After polymerization, the template is removed from the matrix, creating specific recognition sites that are complementary in shape, size, and chemical functionality to the target, conferring to the polymer a kind of permanent memory that will allow it to selectively recognize the analyte from mixtures of similar compounds. They are usually robust and stable in relation to drastic changes of pH, temperature, and solvents, and they are easy to prepare. MIPs can be used as sorbents for solid phase extraction or can constitute the recognition element in the development of a sensor, which needs a transducer, such as electrochemical, calorimetric, piezoelectric, or optical, to transform the binding process into a measurable output signal. Biosensors are chemical sensors where the recognition is based on biochemical or biological elements. The receptor can be composed of distinct molecular units called recognition receptors or, alternatively, it can be a material that includes in its composition recognition sites. Therefore, MIPs can be considered as biomimetic receptors, since these materials can show specificities and affinities perfectly comparable with the natural ones and their advantages, such as chemical and physical stability, making them compatible with several detection conditions and conferring relatively long life times [4]. The use of MIPs as receptors greatly depends on the immobilization methods and different immobilization techniques which have been reported in the literature, such as physical entrapment, adsorption, covalent binding, and cross-linking. 

MIPs based on non-covalent interactions have been mostly used in solid phase extraction and sample concentration for the analysis of CGAs. MIP for CGA was successfully applied for the extraction of CGA in the leaves of *Eucommia ulmodies* using methacrylic acid as a functional monomer, divinylbenzene as a crosslinker, and methanol or dimethylsulfoxide as a co-solvent. The prepared MIPs were microspheres with a narrow particle size distribution. The MIP had a specific molecular-recognition ability for CGA, while other related compounds, such as caffeic acid, gallic acid, protocatechuic acid, and vanillic acid, could not be recognized by the MIP [5]. Another approach was the development of a modified bisphenol A molecularly imprinted polymer sorbent used in the hollow fiber solid phase microextraction of CGA. The pre-polymerization solution containing the template was introduced into the polypropylene hollow fiber segment for in situ polymerization. The system was used for selective extraction of CGA in *Echinacea purpurea*, a medicinal plant. The obtained solid phase microextraction (SPME) device exhibits excellent characteristics, such as high porosity and chemical stability, with a limit of detection of 0.08 ng/mL [6].

A rapid and accurate approach was established for simultaneous purification of theophylline and CGA in green tea by coupling hybrid molecularly imprinted solid-phase extraction with HPLC. Satisfactory extraction recoveries for theophylline (96.7%) and CGA (95.8%) were afforded, and the detection limits were 0.01 μg/mL for theophylline and 0.05 μg/mL for CGA [7].

In the same way, some other MIP-based electrochemical sensors have been developed for CGA detection [8,9,10,11,12]. For instance, a sensitive molecularly imprinted electrochemical sensor was constructed by deposition of a molecularly imprinted siloxane (MIS) film. The MIS-imprinted film was electrochemically characterized using differential pulse voltammetry. The MIS/Au sensor was tested in a solution of the CGA template and other similar molecules, showing excellent selectivity towards CGA when compared with structurally similar molecules. The detection limit was 1.48 × 10^−7^ M, and the sensor was successfully applied for the determination of CGA in coffee and tea samples [12].

Most recently, a promising technique for the enrichment and determination of chlorogenic acids from herbal medicines was proposed. The system consists of a solid-phase extraction adsorbent based on MIPs made of 4-vinylpyridine (4VP) as the functional monomer and hydrogenated 5-CQA (H-5-CQA) as the mimic template. Selective extraction and determination of three isomers of caffeoylquinic acids (CQAs), considered as quality markers for *Lonicera japonica* and *Lianhua qingwen* granules, was successfully achieved. The adsorption isotherms, kinetics, and selectivity of the MIPs were systematically compared with those of non-molecularly imprinted polymers. The MIPs showed high selectivity towards chlorogenic acid, cryptochlorogenic acid, and neochlorogenic acid. A procedure using molecularly imprinted solid-phase extraction, coupled with high-performance liquid chromatography, was established for their determination. The recoveries of the chlorogenic acids were found from 93.1% to 101.4% [13].

Although there are a few examples of MIPs for chlorogenic acid recognition present in the literature, not one is based on optical fluorescence, which is very interesting for its great sensitivity in comparison to other spectroscopic techniques, allowing the detection of particular compounds and analytes in low concentrations. Fluorescence sensors based on MIPs can be developed, taking advantage of the fluorescent properties of some of its components, either monomers present inside the polymer or template. Since not all analytes are fluorescent, it is convenient to use fluorescent monomers to have a fluorescent MIP matrix (fMIP) as a recognition element. When the analyte binding occurs, the fluorescent properties changes of the polymer can be measured, and usually a fluorescence quenching, a shift, or even a fluorescence enhancement are observed. Applications of fMIPs as detecting elements are well documented in the literature, such as the detection of cocaine using an optical fiber with a thin MIP film made of acrylamide fluorescein, acrylamide, and ethylene glycol dimethacrylate (EGDMA) [14], or the detection of λ-cyhalothrin in honey samples using allylfluorescein, acrylamide, and EGDMA to build a fMIP layer directly synthetized onto the core shell of Fe_3_O_4_/SiO_2_ particles [15].

More recently, we also developed fluorescent MIPs for the analysis of tyrosol and hydroxytyrosol in aqueous olive leaves extracts using the non–covalent approach [16], as well as for the detection of irinotecan in human plasma using a napthalimide polymerisable derivative (2-allyl-6-[2-(aminoethyl)-amino]napthalimide) as a functional monomer [17].

In the present work, fluorescent polymeric imprinted nanoparticles were synthetized following the non-covalent approach using H-5-CQA **2** as the template. 4VP **3** and a naphthalimide derivative containing a polymerizable vinyl group **7** were used as functional monomers, capable of interacting with the template by relatively weak non-covalent interactions, such as π-staking, hydrogen bonding, hydrophobic interactions, or van der Waals forces (Figure 1).

## 2. Materials and Methods

### 2.1. Chemicals

All chemicals and solvents were purchased from Sigma Aldrich. Crimp cap Weaton vials used for the polymer synthesis were purchased from Sigma Aldrich. Dialysis membrane MWCO 3500 Da was purchased from Spectrumlabs. Polysine slides for polymer immobilization were purchased from Thermo Scientific.

### 2.2. Instrumentation

NMR spectra were recorded on a Varian 400 spectrometer or on a Varian 500 spectrometer using DMSO-d_6_ as the solvent. ^1^H-NMR titrations were performed on a Varian 500 spectrometer. The resonance multiplicity is described as s (singlet), d (doublet), t (triplet), q (quartet), m (multiplet), dd (doublet of doublets), or br (broad signal).

Fluorescence measurements were performed on a Synergy H1 hybrid reader (Biotek) spectrofluorometer and on a Varian-Cary Eclypse spectrofluorometer with a thermostat at 25 °C.

TEM images were recorded with a Camera Olympus QUEMESA and software RADIUS (EMSIS) on a TEM images Philips EM208 at 100 KV using a 200-mesh copper grid with carbon film.

Rebinding tests were performed on a 1290 UHPLC system (Agilent, Germany), consisting of a degasser, a quaternary pump, a thermostated column compartment, and a diode array detector (DAD) operating at 324 nm and 273 nm, and on a HPLC Thermo scientific Vanquish Dionex Softron consisting of a degasser, a quaternary pump, a thermostated column compartment, and a DAD operating at 325 nm and 270 nm.

### 2.3. Synthesis of Mimic Template H-5-CQA

5-*O*-caffeoylquinic acid **1** (200 mg, 0.56 mmol) was dissolved in EtOH (30 mL) under argon atmosphere, and 40 mg of Pd/C 10% was added. The reaction mixture was stirred at room temperature under H_2_ atmosphere for 6 h. Pd/C was removed by filtering the reaction mixture over celite. EtOH was removed under pressure, and the resulting oil was redissolved in water and freeze-dried to afford reduced H-5-CQA **2** as a white solid (181 mg, 95%). NMR data were in accordance with the literature [13].

### 2.4. Recrystallization of AIBN

2.0 g of AIBN was placed in a three-neck bottomed flask equipped with a stirring bar and a condenser. The vacuum and a flow of argon was alternated in the system 3 times to avoid any trace of oxygen. AIBN was dissolved in 5 mL of ethanol, and the temperature was increased to 50–55 °C. At this temperature, 2 mL of ethanol was added to solubilize the product, and the flask was then left to reach room temperature to allow the crystallization.

### 2.5. Synthesis of 4-[(2-ethylenediamine)] N-allyl-1,8-naphthalimide 7

1.0065 g (1 eq) of 4-chloro-1,8-naphthalic anhydride was dissolved in 45 mL of ethanol, and the resulting solution was heated up to 55 °C under continuous stirring; at this temperature, 330 µL of allyl amine (1 eq) and 5 mL of ethanol were added. The mixture was refluxed for four hours. After cooling at room temperature, the solid was filtered, washed with ethanol, and dried under vacuum to give 652 mg (yield 55%) of 4-chloro N-allyl-1,8-naphthalimide as a light brown powder. 

A mixture of 645 mg of 4-chloro N-allyl-1,8-naphthalimide (2.04 mmol, 1 eq), 1.9 mL of ethylene diamine (24.5 mmol, 12 eq), and 40 mL of ethanol was heated under reflux. After 20, 23, 42, and 46 h, a further three equivalents of ethylene diamine were added, and the reaction was stopped after 66 h. Solvent was removed under reduced pressure to obtain a red oil and, subsequently, 60 mL of water was added, and the mixture was cooled overnight at 4 °C. The solid obtained was filtered, washed with cool water, and dried at 60 °C for two days. 553 mg of pure **7** was obtained as a red solid (yield 92%). NMR data were in accordance with the literature. [18]

### 2.6. ^1^H-NMR Titrations

Interactions between the functional monomers and the template molecule were investigated by titrating the functional monomers **3** and **7** with 5-CQA **1**. In general, 4 mM solutions of monomers in DMSO-d_6_ were titrated with increasing amounts of chlorogenic acid **1** (5-CQA) to obtain final concentrations ranging from 2 mM to 40 mM.

### 2.7. Synthesis of Molecularly Imprinted Polymers (General Procedure)

The functional monomers and the mimic template (H-5CQA) (in the amount reported in Table 1) were dissolved in DMSO, corresponding to 99.5% in weight of total functional monomers and crosslinker. (Equation (1)) After stirring for 60 min, the solution was transferred to a crimp cap Wheaton vial and 60% (in mol) of *N,N′*-methylenebisacrylamide (MBA) **4** or divinylbenzene (DVB) **5**, 5% or 10% (in mol, calculated in the amount of the available double bonds) (see Table 2) of recrystallized azobisisobutyronitrile (AIBN), were added. The vial was left first under vacuum and then was flushed with argon (3 times for 5 min). Radical polymerization was achieved after heating the vial up to 70 °C for 24 h. Each polymer was synthesized either in presence of the template molecule (to obtain fMIP) or without the template (to obtain fNIP). The resulting orange solutions were dialyzed against water for 2 days, a mixture of acetic acid and methanol 2:8 for two days, methanol (10%) for 2 days, and against water for one day, changing the solvent 3 times a day. Finally, the solutions were freeze-dried, leading to a fluffy orange polymer for fMIP and a yellow polymer for fNIP.

(1) $$m\left(S\right)=\frac{\left[m\left(fm\right)+m\left(cm\right)+m\left(cl\right)\right]\cdot \%solvent\;in\;mixture}{\%monomer\;in\;mixture\;}$$

where *m*(*S*), *m*(*fm*), *m*(*cm*) and *m*(*cl*) are the mass of the solvent, the functional monomer, the co-monomer, and the crosslinker, respectively.

The concentration of initiator and crosslinker were calculated using Equations (2) and (3), respectively:

(2) n(I)=[n(fm)+n(cm)+2n(cl)]·%iniziator

(3) $$n\left(cl\right)=\frac{n\left(fm\right)+n\left(cm\right)}{\%\left(fm\right)+\%\left(cm\right)}\;\cdot \%\left(cl\right)$$

where *n*(*I*), *n*(*fm*), *n*(*cm*), and *n*(*cl*) are, respectively, the number of moles of the initiator, the functional monomer, the co-monomer, and the crosslinker.

### 2.8. Transmission Electron Microscopy

Lyophilized fMIPs were suspended in distilled water (0.2 mg/mL) and sonicated for 30 min. Each solution was then diluted 40 times with distilled water and sonicated for 1 h. After filtration on a 0.45 μm PTFE syringe filter, a drop of solution was placed on an amorphous carbon coated grid and left to dry at room temperature for one night; TEM images of the MIPs were finally recorded.

### 2.9. Rebinding Tests

A mixture of 2.0 mg of polymer (fMIP or fNIP) and 5-CQA (80 µM) in 2 mL of water was kept under stirring at room temperature. Aliquots of 200 µL were taken at different times between 10 min and 24 h and centrifuged (15,100 rpm for 10 min). The supernatant was analyzed by UHPLC to quantify the residual free template concentration. A 1290 UHPLC system (Agilent, Germany) was used with a Kinetex XB-C18 column 2.6 μm 100 × 2.1 mm (Phenomenex, USA) at 25 °C. Solvents were used at a total flow rate of 0.5 mL/min, and the volume of injection was 2.0 μL. Solvent A was water/formic acid (1000:1 *v/v*), and solvent B was acetonitrile. The gradient profile was from an initial 97% of solvent A to 85% of A in 8 min, then 60% of A at 11 min, and a return to 97% A at 12 min to re-equilibrate. The following wavelengths were used: 324 nm (for 5-CQA) and 273 nm (for caffeine).

The concentration of the free template at each time was calculated as reported in our previous work [16].

### 2.10. Cross Reactivity Tests

The polymer selectivity was investigated following the same approach of the rebinding tests by UHPLC. An amount of 2.0 mg of fMIP01 was added to 2.0 mL of a solution containing a mixture of pure standards of 5-CQA, caffeic acid, *p*-coumaric acid, and caffeine, each at 80 μM concentration. The mixture was incubated at 25 °C under continuous stirring, and 200 μL aliquots were taken at different times (from 10 min to 24 h). Each aliquot was centrifuged (12,000 rpm for 10 min) to remove the polymer, and the supernatants containing an unknown amount of the standards were analyzed by UHPLC.

### 2.11. Fluorescence Titrations

The fluorescence property of fMIP01 was analyzed by fluorescence titrations of a 30 μg/mL solution of the polymer, obtained by dilution in DMSO and water:DMSO (9:1) of the polymer mother solution in DMSO (1 mg/mL), with increasing concentrations of the target molecule 5-CQA from micromolar to millimolar concentrations. The fluorescence emission of the polymers was analyzed at 530 nm and 540 nm. The excitation wavelength was 440 nm.

### 2.12. Analysis on Real Sample

Green coffee beans were ground to a fine powder in a mixer ball mill MM400 (Retsch, Haan, Germany) and extraction was performed in duplicate by dynamic heat-assisted water extraction [19].

For this purpose, 1 g of powdered *Coffea arabica* green coffee from Brazil was added to 100 mL of boiling water, and the mixture was stirred for 10 min at 200 rpm on a heated plate (Arex Velp Scientifica, Usmate, Italy) and filtered.

The filtrate was diluted 1:20 and analyzed by HPLC using a Thermo scientific Vanquish Dionex Softron instrument with a Kinetex C18 250 × 4.6 mm 5 µm 100 Å (Phenomenex) column, a column guard, a 100 µL loop, and a UV detector set at 325 nm (for 5-CQA) and 270 nm (for caffeine). The flow was set to 1 mL/min and injections were of 20 μL. The eluent was: solvent A H_2_O + 0.05% TFA and solvent B CH_3_CN + 0.05% TFA in a 85:15 ratio for the calibration curve of 5-CQA (Figure S1) and caffeine (Figure S2) (see Supplementary Material), while a gradient was used for the analysis of coffee extract (10 min 85% A and 15% B, from 15% B to 60% B in 20 min, from 60% B to 15% in 10 min, and 10 min 85% A and 15% B). Standard stock solutions were prepared in water in the range of concentration 50–250 µM for calibration curve of 5-CQA and caffeine. Each solution was analyzed by HPLC in triplicate, and the mean value of each solution was used for the calibration curve (Figures S1 and S2). The LOD and LOQ resulted in 6.4 μM and 19.5 μM, respectively. They were calculated according to the following equations:

LOD 3.3 × σ/S and LOQ 10.0 × σ/S, where σ is the standard deviation and S is the slope.

### 2.13. Immobilization Test

80 μL of 10% solution of glutaraldehyde in PBS was added to different commercial polysine^®^ slides and left at room temperature for one hour. Subsequently, the glass plates were washed with 3 mL of water (1 mL at a time). A mixture was prepared by mixing a 20 mg/mL gelatin solution in water and 100 μg/mL suspension of fluorescent polymer MIP01 in water:DMSO (9:1) in a ratio 1:1, and 80 μL of the mixture was added to different polysine^®^ slides. These glass plates were kept in the dark at room temperature for 24 h. Then, the fluorescence of the immobilized polymer on the plates was measured before and after washing with 3 mL of water (1 mL at a time) to determine the residual concentration of the immobilized polymer. Soon after, 40 μL of solutions of a range of concentrations between 78 μM and 80 mM of a 5-CQA pure standard solution was added to the different plates in duplicate, and the samples were incubated for 90 min in the dark at room temperature. Finally, the fluorescence intensity of the polymer in each plate was measured before and after washing them with 2 mL of water (500 μL at a time).

## 3. Results and Discussion

### 3.1. ^1^H-NMR Titrations

To achieve polymers suitable for the selective identification of the analyte, it is necessary to preliminarily evaluate the interactions between the analyte and the monomers in order to establish which are the best monomers to be used. Interactions can be observed by ^1^H-NMR titrations and the specific functionalities that are involved in the formation of the functional monomer–template complex before polymerization. It is known that the degree of template–monomer complexation is directly dependent upon the types of interactions employed and the chemical composition of the polymerization reaction mixture [20]. 4-Vinylpyridine (4VP) has already been used as a functional monomer for interactions with 5-CQA and is one of the most common functional monomers used for the preparation of MIPs due to its commercial availability and low cost [21,22]. 4-[(2-ethylenediamine)]-*N*-allyl1,8-naphtalimide **7** is a fluorescent molecule synthesized from 4-chloro-1,8-naphtalic anhydride [18], and its analogues have been widely used in the literature to obtain fluorescent polymers to be used in sensor development [16,23]. The interactions between the template and each functional monomer were studied by ^1^H-NMR in DMSO-d_6_ by progressive addition of a solution of 5-CQA to a solution of the functional monomer in order to evaluate the variations of the chemical shift of the single protons of the monomer. In the Supplementary Material, the histograms of the chemical shift variations in the ^1^H-NMR spectrum of 4VP upon progressive addition of 10 equivalents of 5-CQA, as well as selected changes of NMR peaks upon titrations for the most relevant cases (Figures S3 and S4), are reported. In this way, depending on the downfield or upfield chemical shift, it is possible to identify protons involved in the interactions and the type of the interaction [24].

The addition of 5-CQA to 4VP solution causes a progressive shift of all signals. The variation of the proton chemical shift (δ_final_–δ_initial_) of the monomer 4VP after addition of 10 equivalents of template is shown in Figure S3. Variations of the aromatic protons of the pyridine ring towards downfield are evident (Figure S4).

In the addition of 5-CQA to the functional monomer **7**, protons of CH_2_NH_2_ showed a greater shift towards downfield than the others, probably due to a proton exchange in the acid–base reaction between the amino group of **7** and the carboxylic group of 5-CQA. (Figure S5) In Figure S6, it is possible to observe, specifically, the trend of the titration curve of the protons adjacent to the amino group of monomer **7**. The plateau is reached after the addition of approximately two equivalents of 5-CQA, which represents the saturation point. In order to evaluate the behavior of the template in the presence of both monomers, a ^1^H-NMR titration was carried out by adding increasing amounts of the template to a mixture of monomers **3** and **7** (Figure S7). As it can be observed in Figure S7b, a first plateau is reached by the aromatic proton of 4VP **3** after 0.5 eq of 5-CQA addition, but when increasing the template concentration, the chemical shift linearly increases to reach a second plateau after eight equivalents, in accordance with hydrogen bond formation between the nitrogen atom of the pyridine and the hydroxyl groups of the phenol ring. Alternatively, a π–π stacking interaction between aromatic rings could explain an upfield shift of the aromatic protons. Anyway, the main interaction of 5-CQA occurs with monomer **7**. The downfield shift of the protons CH_2_NH_2_ indicates a deshielding effect due to the positively charged NH_2_ due to the hydrogen transfer from the carboxylic group.

Both functional monomers showed a significant interaction with 5-CQA and could contribute to the efficiency of the fMIP to recognize 5-CQA and were used in the synthesis of fMIPs.

### 3.2. Synthesis and Characterization of Molecularly Imprinted Polymers

fMIPs were synthesized under high dilution radical polymerization conditions following a standard protocol [25]. By optimization of solvent and monomers concentrations, it was possible to obtain polymer particles of nanometric dimensions. In this way, interactions between the template and functional monomers are favored thanks to high surface area of the particles, allowing the formation of more accessible binding sites in a short diffusion time [26]. The choice of a suitable solvent allows the formation of highly stable nano gels due to the high viscosity of the colloidal solutions. The solvating power of the solvent prevents macrogelation via osmotic repulsion forces and steric hindrance, without adding surfactants, and, furthermore, under diluted conditions in a suitable solvent, each polymeric particle is stabilized, avoiding intermolecular crosslinking [27].

To avoid aggregation and macrogelation of the polymer, it is important to keep the concentration of all monomers under a critical value, called critical monomer concentration (C_M_). The C_M_ is defined as the percentage by weight of all monomers used for the polymerization, as compared to the percentage of the overall mass of monomers and solvent used for a polymer preparation [28]. For our purposes, DMSO was chosen as the solvent for polymer preparation due to its ability to dissolve all reagents involved in the synthesis.

**Figure 2:**
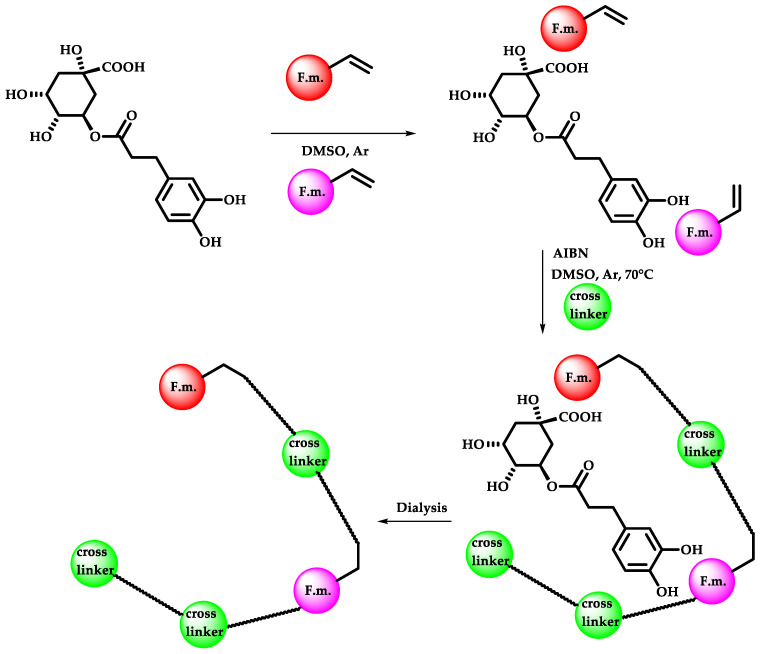
The three steps for the synthesis of fMIPs.

fMIP preparation requires three steps, which are represented in Figure 2: pre-polymerization (step I), radical polymerization (step II), and removal of the template (step III).

In the pre-polymerization step, a mixture of the functional monomers and the template in DMSO was kept under stirring for 1 h at room temperature in order to favor the formation of a complex functional monomer–template. The ratio used between the functional monomer and the template was fixed at 1:1.2. A little excess of the template was used in order to obtain a high number of specific binding sites, assuming a 1:1 monomer:template interaction. The aim of this step is to favor the formation of monomer–template complexes through non-covalent bonds, allowing the pre-formation of the binding sites that will be retained after the polymerization phase.

In the radical polymerization the co-monomer (if present), the crosslinker MBA or DVB and the radical initiator AIBN were added to the solution. The concentration of the crosslinker was fixed to 60%. The use of high percentages of crosslinker is essential to impart the necessary rigidity to preserve the shape and stability of the binding sites formed inside the polymeric matrix. However, polymers with a crosslinker content above 90% lead to too rigid and less flexible polymers [29]. The amount of radical initiator was fixed initially at 10% and was successively used at 5% based on the results obtained. The polymerization was carried out at 70 °C for 24 h.

In the last step, the removal of the template was accomplished by dialyzing the polymers in water, then in methanol:acetic acid (8:2), followed by water:methanol (90:10), and, finally, in water. The use of methanol increases the solubility of template molecules and leads to a polymer shrinkage that forces the dissociation of the MIP–template complex, while water allows one to remove the unreacted functional monomers or by-products and to resume the better polymer conformation [30].

At the beginning, the polymerization reaction, using 5-CQA as template, was conducted in DMSO-d_6_ to verify, via ^1^H-NMR, the possible incorporation of the template into the polymer due to the double bond present in the cinnamoyl moiety. (Figure S8) In the meanwhile, it was also possible to determine the amount of unreacted monomer after polymerization using a known amount of syringic acid, which was added to the reaction mixture as the internal standard.

From the results obtained, we observed that around 70% of 5-CQA was polymerized, while 4VP and MBA were almost completely polymerized, and monomer **7** was incorporated at 55%. (Figure S9) Due to the high percentage of polymerization for 5-CQA, it was necessary to use a mimic template, which could not be incorporated into the polymer. Hydrogenation with hydrogen on Pd/C of the double bond of 5-CQA furnished H-5-CQA, which was prepared following the literature procedure [13].

As a second attempt, H-5-CQA was used as the mimic template, and by ^1^H-NMR analysis, it was determined that the mimic template H-CQA was completely absent in the polymer structure, thus confirming that hydrogenation of 5-CQA is necessary. Moreover, in this case, 4VP and MBA were quite completely incorporated inside the polymer, while monomer **7** was incorporated at only 40%.

According to the results obtained in DMSO-d_6_, four different fMIPs have been synthetized using the composition reported in Table 2. In the synthesis of MIP02, the co-monomer NIPAM was also introduced, since it is capable of modulating the solubility of the polymer in water. As it has been reported in the literature, it does not interact with the template agent in polar solvents such as water or DMSO, but limits itself to acting as an inert and stabilizing component of the polymeric structure [31]. In fMIP04, the DVB **5** crosslinker was also considered, which was used in a previous work with excellent results, in order to enhance the interactions with the aromatic ring of the template by π–π stacking [16].

For each polymer, a non-imprinted polymer (fNIP) was also prepared as a control polymer using the same polymerization procedure and the same concentrations of monomers, but without the presence of H-5-CQA as the mimic template.

**Figure 3:**
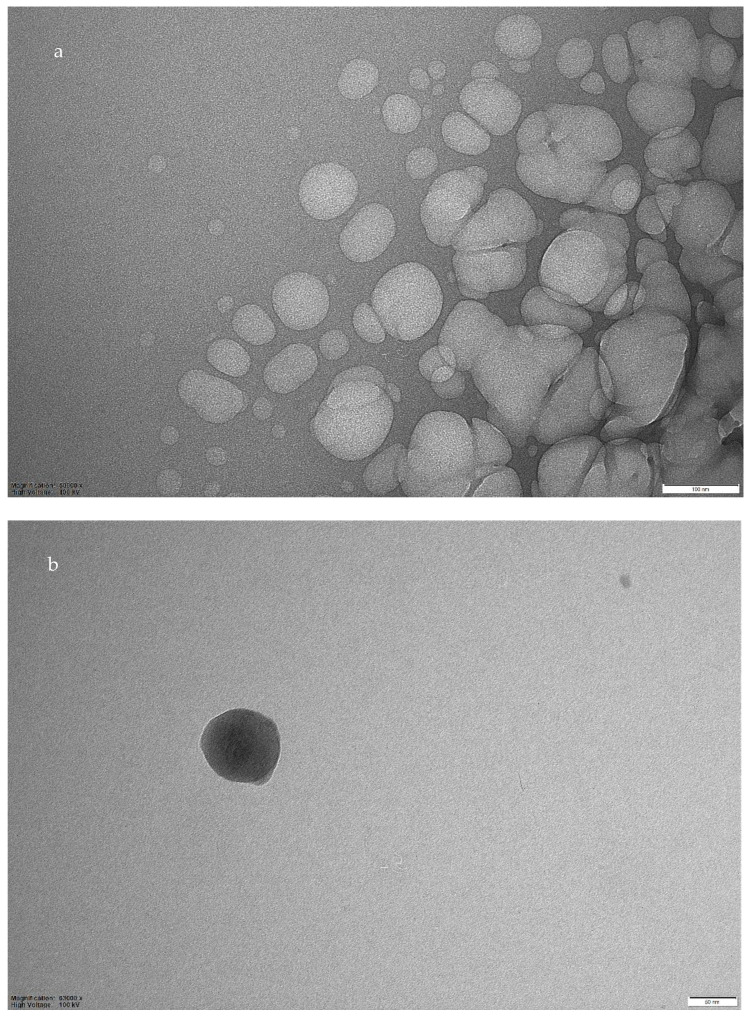
TEM images of fMIP03. (**a**): enlargement 50,000×, bar 100 nm; (**b**): enlargement 63,000×, bar 50 nm.

TEM analysis was performed on MIP03, and aggregates are visible in Figure 3a, with a size of about 2 μm. Isolated particles are also present, with sizes in the range 45–90 nm (Figure 3b).

This tendency of the polymer to aggregate can be due to the presence of 4VP, as well as due to other polar groups in the polymeric matrix, since the formation of aggregates in polymers containing 4VP have already been reported in the literature [16,17].

### 3.3. Rebinding Tests

**Figure 4:**
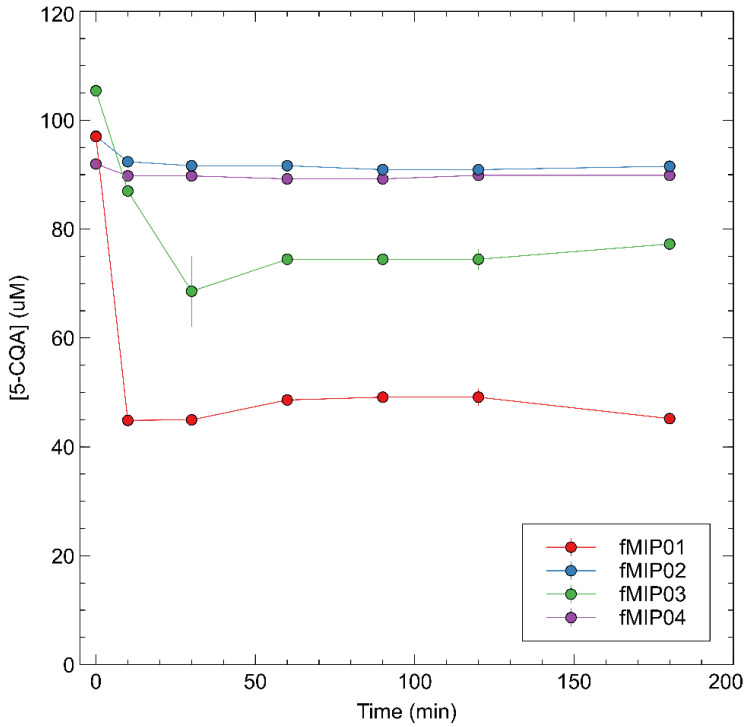
Rebinding kinetics of 5-CQA with fMIP01, fMIP03, fMIP03, and fMIP04.

**Figure 5:**
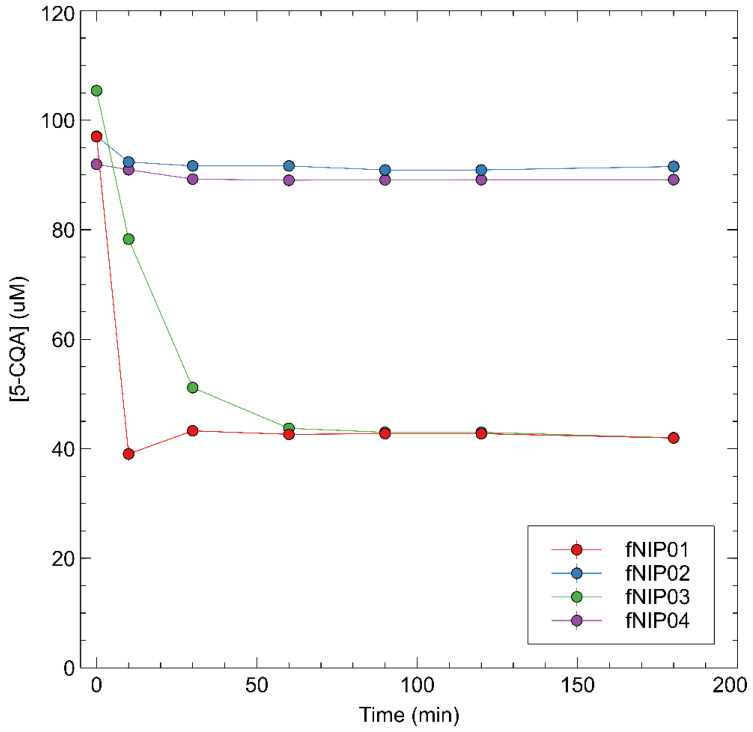
Rebinding kinetics of 5-CQA with fNIP01, fNIP03, fNIP03, and fNIP04.

From rebinding tests, fMIP01 showed a good affinity for 5-CQA, binding 48 nmol after a few minutes of incubation and achieving the saturation point after 90 min of binding 70 nmol of 5-CQA. However, fNIP01 did not show any significant differences with respect to the binding affinity of fMIP01. This tendency could probably indicate that the interactions between functional monomers and templates occur in a very similar way, between both imprinted and non-imprinted polymers, with a prevalence for the non-specific interactions. Other molecularly imprinted polymers reported in the literature, using a mimic template, have shown similarly favorable performances between MIPs and NIPs, indicating that the imprinting binding sites play a minor role in selective target detection [32] (Figure 4 and Figure 5).

fMIP03, which differs from fMIP01 for the amount of AIBN used in the synthesis, shows even higher discrepancies between fMIP and fNIP rebinding capacity, with the fNIP able to capture more 5-CQA than the corresponding fMIP. Anyway, the capacity of rebinding 5-CQA is lower with respect to fMIP01, which indicates that an increasing amount of AIBN determines the formation of even more non-specific sites inside the polymer. fMIP04 and fMIP02 did not show any ability to capture the template, showing that both DVB as a cross-linker and NIPAM as a co-monomer do not enhance the performance of the polymer, but lead to a loss of efficiency.

### 3.4. Cross Reactivity

The selectivity was determined for only fMIP01 and fNIP01, which gave the best results in rebinding tests and was performed on a mixture of the analyte 5-CQA, together with the free hydroxycinnamic acids, such as caffeic and *p*-coumaric acid, as well as with caffeine. The tests were carried out treating a solution of 1 mg/mL of polymer with a mixture of 80 μM of each pure standard of 5-CQA, caffeic acid (CA), *p*-coumaric acid (*p*CoQA), and caffeine (CAF). The remaining standard concentrations in solution were measured by UHPLC after different incubation times.

As observed from Table 3, fMIP01 was able to capture 5-CQA and CA and *p*-CoQA to a minor extent, while it was unable to bind caffeine. The cinnamic part of the 5-CQA is formed by CA, which only differs from *p*-CoQA structure by the presence of two phenolic hydroxyl groups instead of one, so these molecules could have access to the binding sites of the polymeric matrix and can be recognized by the polymer.

When comparing the cross reactivity of fMIP01 and fNIP01 regarding the different targets, it can be noticed, from Table 3, that fNIP01 is selective only towards caffeine, while the imprinting effect is clearly present in fMIP01 due to the presence of specific binding sites capable of recognizing the analytes that are absent in fNIP01. For this reason, the fluorescence changes of the imprinted polymer when interacting with the template were evaluated, and the results will be discussed below.

### 3.5. Rebinding on the Real Sample

**Figure 6:**
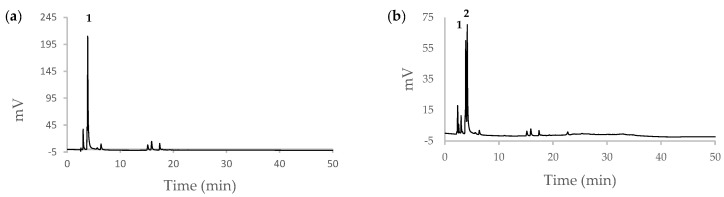
HPLC chromatograms of coffee extract (**a**) UV at 325 nm, peak 1: 5-CQA; (**b**) UV at 270 nm, peak 1: 5-CQA, peak 2: caffeine.

**Figure 7:**
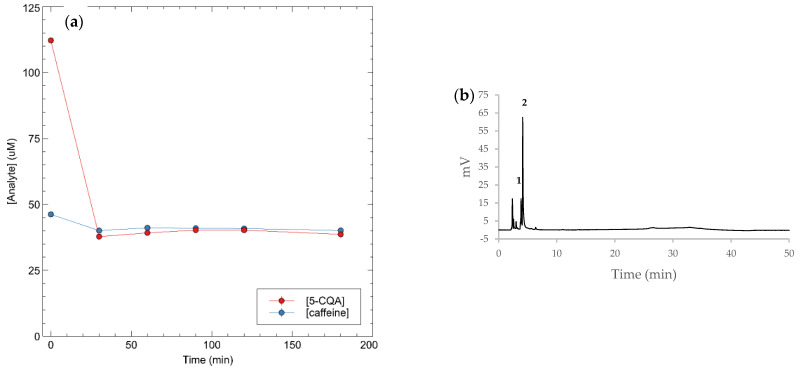
(**a**) Rebinding kinetics of 5-CQA and caffeine in coffee extract; (**b**) HPLC chromatogram of coffee extract with fMIP01, UV at 270 nm, peak 1: 5-CQA, peak 2: caffeine.

Green coffee beans of *Coffea arabica* were extracted with boiling water, and the contents of 5-CQA and caffeine were determined by HPLC using a calibration curve for 5-CQA (detecting at 325 nm) and for caffeine (detecting at 270 nm). fMIP01 was tested on a 1:20 diluted sample of coffee extract, and the HPLC chromatograms are reported in Figure 6a,b. In Figure 6b, the presence of caffeine (peak 2) is evident, while, in Figure 7b, for the real sample with fMIP01, it is evident that fMIP01 is able to selectively capture only 5-CQA since peak 2, relative to caffeine, was not decreasing, while peak 1, relative to 5-CQA, was much less intensive with respect to caffeine.

### 3.6. Fluorescence Titrations

The fluorescent properties of polymer fMIP01 were evaluated by studying the interactions between fMIP01 and the target molecule (5-CQA). Changes in the polymer fluorescence at low concentrations were registered by fluorescence titration of the polymer, with increasing amounts of the analyte in two different solvents: DMSO and water:DMSO (9:1).

**Figure 8:**
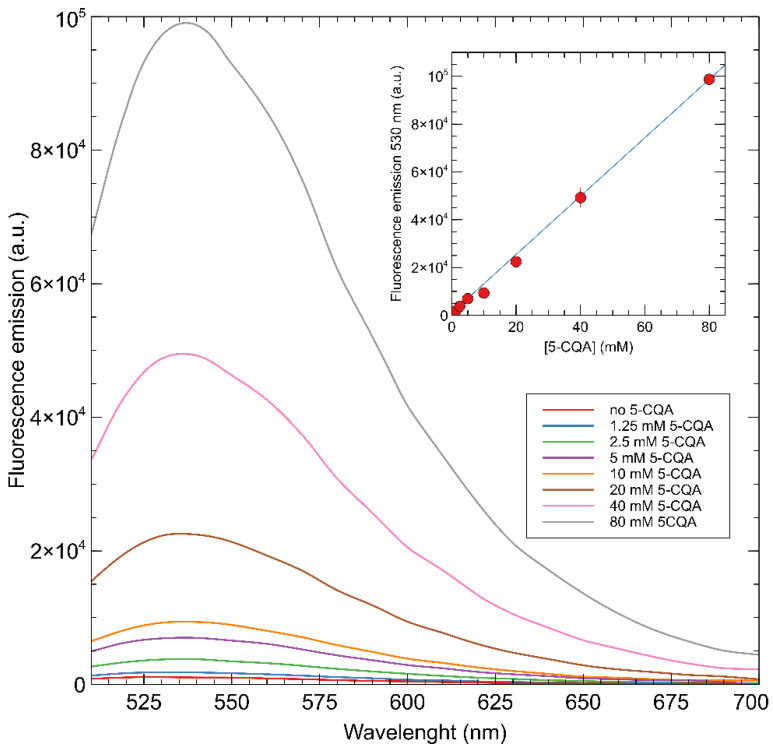
Fluorescent emission titration spectra of fMIP01 in DMSO after addition of 5-CQA.

As a first choice, titrations were carried out by adding increasing amounts of 5-CQA from micromolar to millimolar concentrations to a solution of 30 μg/mL of fMIP01 in DMSO. In Figure 8, we can observe a three-fold increase in fluorescent intensity at the emission maximum of 530 nm by addition of the analyte. The fluorescence enhancement is probably due to the hydrogen transfer between the carboxilic group of the analyte and the amino group of the monomer **7** present in fMIP01, making PET (photoinduced electron transfer) communication between the receptor and the fluorophore not thermodinamically favored, and it becomes cut off, since the lone pair of electrons of the receptor are no longer available after recognition of the analyte. As it has been reported in the literature, in these types of “off-on” systems, that protons from the analyte electrostatically attract the electrons, thus increasing the oxidation potential of the analyte-bound receptor [33,34].

**Figure 9:**
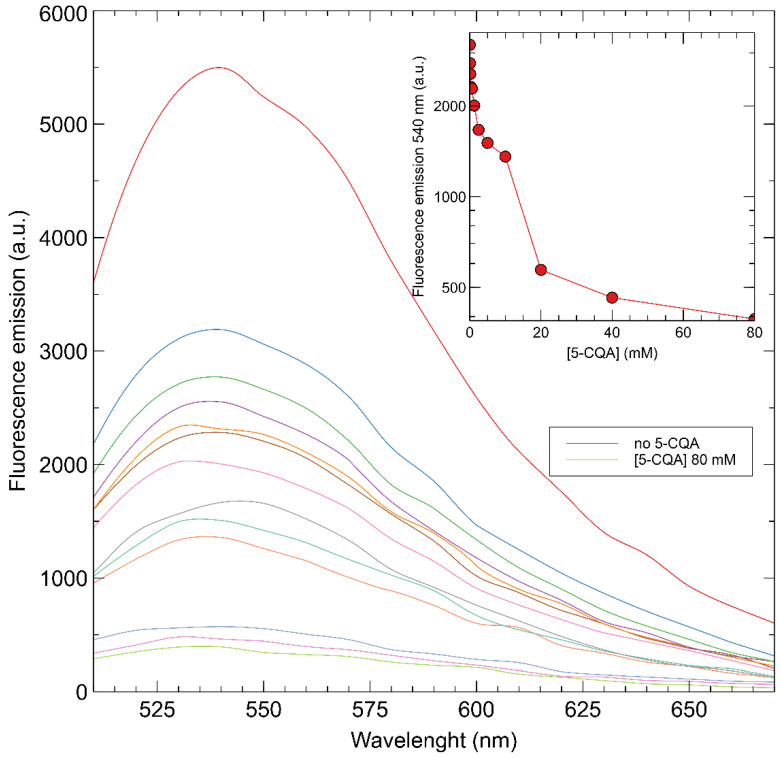
Fluorescent emission titration spectra of fMIP01 in water:DMSO (9:1) after addition of 5-CQA.

Changing the solvent from DMSO to a mixture of H_2_O/DMSO 9:1 (at a pH of 7.7), a bathochromic shift with respect to the system in DMSO was observed, with the emission maxima registered at 540 nm instead of 530 nm. This effect could be a consequence of the π→π* internal electron transfer transitions (ITC) that have already been observed for these types of dyes when the polarity of the solvent increases [18]. Moreover, a quenching of the fluorescence was instead observed (Figure 9), showing a decrease in fluorescence of 58% by adding a 39 μM solution of the analyte, while a total quenching of 92% was obtained at a final concentration of 80 mM of the target molecule.

A possible explanation for the quenching is that, in presence of water, the amino group of the fluorescent monomer can be protonated, cutting off the PET process, which means that the system has been switched “on” prior to the recognition event.

**Figure 10:**
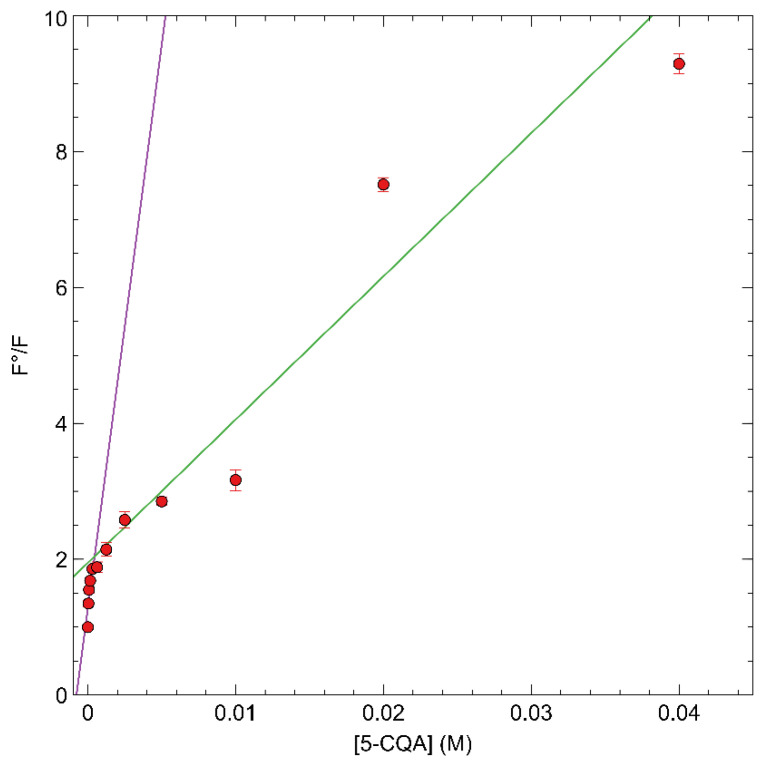
Stern-Volmer plot of fMIP01 for 5-CQA.

The Stern-Volmer plot obtained with fMIP01 is reported in Figure 10, where a bimodal quenching behavior can be noticed with a first, higher slope, linear region at low concentrations of 5-CQA and a second, lower slope, linear region at higher 5-CQA concentrations. This behavior can be an indication of the presence of non-homogeneous binding sites inside the polymers with different affinity for 5-CQA. 

The efficiency of the analyte in quenching the fMIP01 emission was investigated by the Stern-Volmer Equation (4), [15] which was separately applied on the two linear regions of the Stern-Volmer plot (Figure 10).

(4) $$\frac{F_{0}}{F}=K_{app}^{SV}\cdot \left[5-CQA\right]+1$$

The bimolecular quenching constant *K_q_^app^* can be calculated by Equation (5) [35]:

(5) $$K_{q}^{app}=\frac{K_{SV}^{app}}{\tau_{0}}$$

*K_q_^app^* was calculated using the lifetime (*τ_0_* ) of an analogue 4-amino substituted naphtalimide monomer used to develop fMIPs to respond to carboxylate-containing guests. The reported *τ*_0_ of this molecule in DMSO corresponds to 0.11 ns [36]. From Equations (4) and (5), the values of *K^app^_sv_* and *K_q_^app^* for fMIP01 were 2.11 × 10^2^ L·mol^−1^ and 1.9 × 10^12^ L·mol^−1^s^−1^, respectively, at higher concentrations, while they were 1.6 × 10^3^ L·mol^−1^ and 1.5 × 10^13^ L·mol^−1^s^−1^, respectively, at lower concentrations.

According to the literature, quenching constants (*K_q_*) with values below 1 × 10^10^ L·mol^−1^s^−1^ are found for dynamic interaction mechanisms, while higher values of *K_q_* suggest that a static mechanism is taking place [35,37]. In our case, since *K_q_^app^* for fMIP01 is higher than the limit value for the diffusion-controlled quenching (1 × 10^10^ mol^−1^L^−1^s^−1^), the observed quenching of the polymer fluorescence, with the presence of the analyte, can be considered as the result of a static interaction, and *K^app^_SV_* can be considered as an apparent association constant between the population of fluorophores in the MIP and the quencher.

### 3.7. Immobilization of fMIP01

Since physical entrapment of MIPs into gels or membranes has been previously reported in the literature for their use as electrochemical transducer, [38] the fluorescent polymer fMIP01 has been physically entrapped into a gelatin gel covalently bonded to a functionalized support.

**Figure 11:**
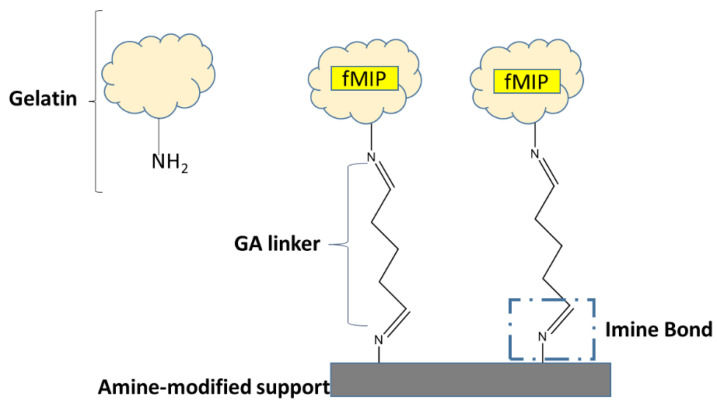
Schematic representation of fMIP immobilization.

The entrapment has been carried out using a suspension of 100 μg/mL of fMIP01 in water:DMSO (9:1) and a solution of 20 mg/mL of gelatin, which were mixed together in a ratio of 1:1 and immobilized on a poly-lysine glass support using glutaraldehyde (GA) as a bifunctional crosslinking agent (Figure 11).

Gelatin is a bio-macromolecule obtained by the hydrolysis of collagen, mostly from skin and the connective tissue of animals. Because of the gel-forming property of gelatin at around 35 °C, and its versatility in the amino acid composition, it has been widely used, not only as a stabilizer, emulsifier, and clarifying agent, but also as a protective coating material [39,40]. Moreover, due to its low cost and efficiency in the stabilization of collagenous materials, GA is the most widely used cross-linking molecule able to form a Shiff base by reaction of non-protonated amino groups (-NH_2_) of lysine to the carbonyl groups (C=0) of the aldehyde. The mechanism of the covalent bond formation between the gelatin and the GA is greatly dependent upon pH conditions, and it has been extensively reported in the literature [39]. The new covalent bonds can be either intramolecular (short-range) or intramolecular (long-range), which are the result of the polymerization of glutaraldehyde or aldol condensation reaction.

The residual immobilized concentration of the fluorescent polymer was estimated by calculating the ratio between the initial fluorescence intensity and the fluorescence intensity after washing the glass plates with water at 540 nm of different polymers solutions immobilized on fifteen different poly-lysine plates in different times. Mean values are reported in Table 4 and, as can be noticed, about 70% of the initial polymer concentration remains on the plates.

**Figure 12:**
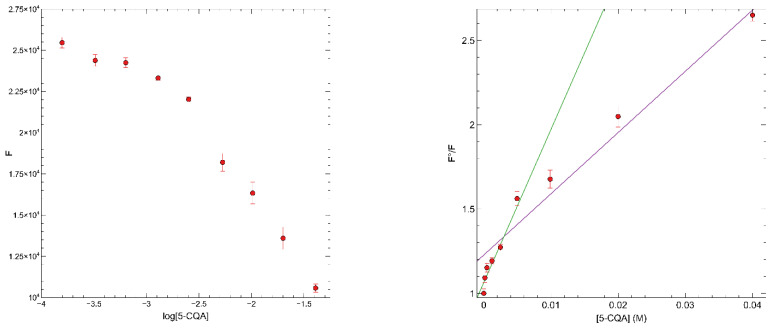
Preliminary calibration curve of immobilized MIP01 with 5-CQA (**left**), Stern-Volmer plots of immobilized fMIP01 for 5-CQA (**right**, green line: regression of low concentration data; magenta line: regression of high concentration data).

Once the polymer was immobilized, the fluorescence properties of the polymer were evaluated by carrying out titrations, in duplicate, with increasing concentrations between 78 μM and 80 mM of a standard solution of 5-CQA. The fluorescence emission of the fMIP01 was measured at 540 nm when excited at 480 nm, before and after incubation, for 90 min. The fMIP01 fluorescence resulted in a correlated quenching in a range from 156 μM to 40 mM of the analyte (Figure 12).

From the slopes of the equation in the Stern-Volmer plot at high and low concentrations, the apparent quenching constants were calculated, as described in the previous section, and the obtained values are 3.5 and 8.6 × 10^11^ L·mol^−1^s^−1^, respectively. Although these values indicate that a static quenching is taking place, the significantly lower values of the apparent Stern-Volmer constants (39 and 91 L·mol^−1^), with respect to the ones obtained by carrying out the titrations in solution (2.11 × 10^2^ and 1.6 × 10^3^ L·mol^−1^), indicate a lower affinity of the polymer for 5-CQA. The decrease is particularly significant for the fraction of high affinity binding sites, which are most likely less accessible than the low affinity ones. This result could be a consequence of some diffusion kinetic problems due to the difficulty of depositing a uniform layer of polymer on the glass surface [41]. For this reason, it is important to consider parameters, such as the concentration of gelatin and crosslinker, as well as the ratio gelatin: polymer, which must be carefully optimized in order to improve the fMIP affinity toward the target molecule to afford a better sensitivity. However, although these first attempts could not be optimized at the moment, the results obtained open the possibility to use fMIP01 as a recognition element in an optical sensor for determining the 5-CQA concentration.

Anyway, the results obtained, which are not optimized, are comparable with those described in the only paper reporting a MIP-based sensor for chlorogenic acid, based on a modified pencil graphite electrode with molecularly imprinted polypyrrole (MIPpy) synthesized by electropolymerization of pyrrole monomer at constant potential in the presence of chlorogenic acid (CGA) [11].

## 4. Conclusions

Four different fMIPs were designed and synthetized and tested for the recognition of 5-CQA. Only fMIP01, containing 4VP and the naphtalimide derivative as monomers, gave satisfactory results in rebinding of 5-CQA, as demonstrated by rebinding kinetics at HPLC. For the real sample, fMIP01 showed a good selectivity, recognizing only 5-CQA and not caffeine. A good response to the fluorescence quenching was also obtained in the range between 39 μM and 80 mM. To demonstrate this, a promising system that can be exploited in the design of an optical sensor for CGA detection is proposed. Polymer fMIP01 was immobilized by physical entrapment on a functionalized glass surface, showing a quenching of fluorescence with an increase in the 5-CQA concentration between 156 μM and 40 mM.

## Figures and Tables

**Table 1:** Composition of the polymerization mixtures.

Polymer	Template 2 [mg]	Functional Monomer 3 [mg]	Functional Monomer 7 [mg]	AIBN [mg]	Co-Monomer 6 [mg]	Crosslinker 4 [mg]	Crosslinker 5 [mg]	DMSO [mL]	Yield %
fMIP01	16.0	4.7	13.3	3.0	-	20.8	-	7.05	40
fNIP01	-	4.7	13.3	3.0	-	20.8	-	7.05	23
fMIP02	8.0	-	6.6	3.0	7.6	20.8	-	6.33	12
fNIP02	-	-	6.6	3.0	7.6	20.8	-	6.33	4
fMIP03	16.0	4.7	13.4	6.0	-	20.8	-	7.00	42
fNIP03	-	4.9	13.4	5.9	-	20.9	-	7.00	60
fMIP04	16.0	4.7	13.4	6.0	-	-	17.6	6.40	10
fNIP04	-	4.7	13.2	6.0	-	-	17.6	6.40	15

**Table 2:** Polymer composition.

Polymer	Functional Monomer 3	Functional Monomer 7	Comonomer 6	Crosslinker 4	Crosslinker 5	AIBN
fMIP01	20%	20%	-	60%	-	5%
fMIP02	-	10%	30%	60%	-	5%
fMIP03	20%	20%	-	60%	-	10%
fMIP04	20%	20%	-	-	60%	10%

**Table 3:** Cross reactivity for fMIP01 and fNIP01.

	5-CQA ^a^ (%)	CA ^b^ (%)	*p*CoQA ^b^ (%)	CAF ^b^ (%)
fMIP01	100	88	72	0
fNIP01	100	100	90	0

^a^ the amount of captured analyte was fixed at 100%; ^b^ % fraction of captured analyte with respect to 5-CQA.

**Table 4:** Fluorescent intensities of immobilized MIP01.

Initial MIP Concentration (μg/mL)	F intensity before Washing (STD)	F Intensity after Washing (STD)	Immobilized MIP Concentration (μg/mL)
100	39,453.17 (6437.04)	27,941 (5766)	71

## Data Availability

Not applicable.

## Supplementary Materials

The following supporting information can be downloaded at: https://www.mdpi.com/article/10.3390/s22249874/s1, Figure S1: HPLC calibration for 5-CQA; Figure S2: HPLC calibration for caffeine; Figures S3–S7: chemical shift variations for monomers at ^1^HNMR; Figures S8 and S9: NMR spectra before and after polymerization; Figures S10 and S11: TEM images.
