# Supplementary material for: Fluorescent Imprinted Nanoparticles for Sensing of Chlorogenic Acid in Coffee Extracts
Source: Sensors (Basel). 2022 Dec 15;22(24):9874. doi: 10.3390/s22249874 (PMC9785805; doi:10.3390/s22249874)
Supplement: Supplementary file 1 [file sensors-22-09874-s001.zip › sensors-2082258-supplementary.pdf]

Fluorescent Imprinted Nanoparticles for Sensing of chlorogenic acid in coffee extracts

Anggy Lusanna Gutierrez-Ortíz[1], Veronica Vida[1], Matjaz Peterka[2], Jasmina Tušar[2], Federico Berti[1], Luciano Navarini[3], Cristina Forzato [1,*]

Supplementary Material

Calibration curves

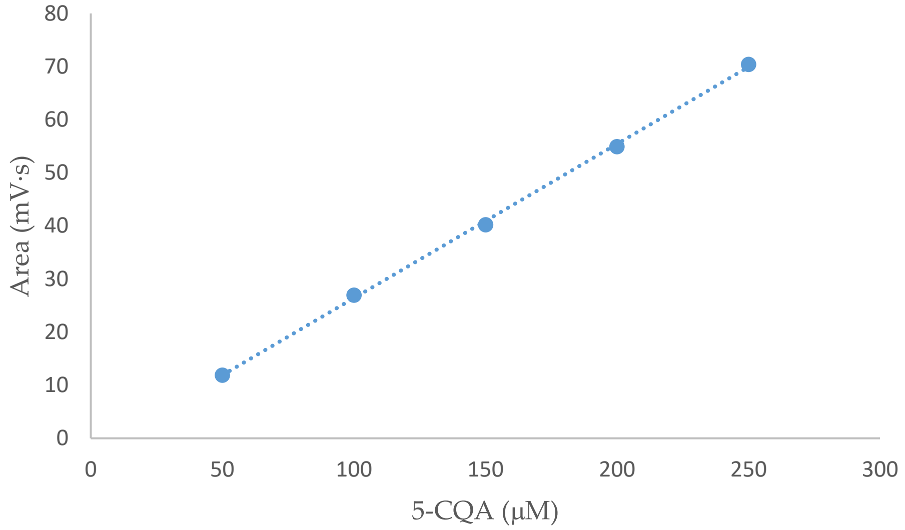

Figure S1. HPLC calibration for 5-CQA, average of triplicate measures. Linear regression for 5-CQA: r^2 0.999, slope 0.29, intc. 2.61. Linear regression for HT: r^2 0.997, slope 47.01, intc. -29.15

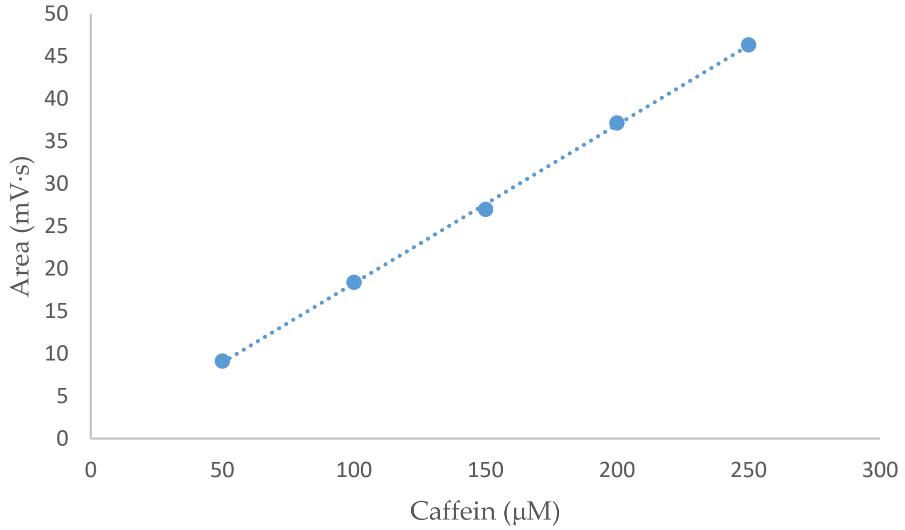

Figure S2. HPLC calibration for caffeine, average of triplicate measures. Linear regression: r^2 0.999, slope 0.19, intc. 0.37

Interactions of functional monomers with the targets: NMR titrations

In figures S3 – S7 are reported the most significant changes in the NMR spectra of 4VP and monomer **7** upon titration with increasing amount of 5-CQA leading to the most relevant effect.

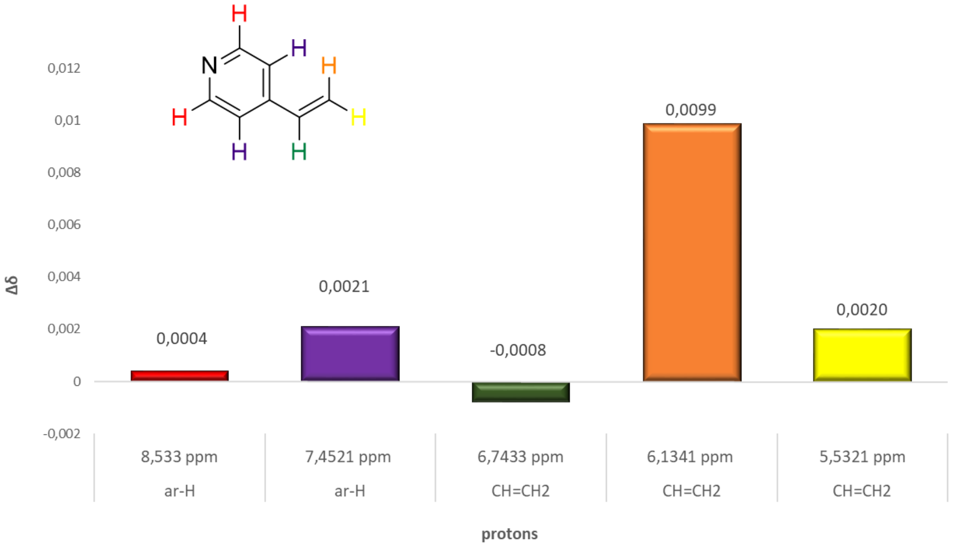

Figure S3. Histogram of the chemical shift variations in the ^{1}H-NMR spectrum of 4VP upon addition of 10 equivalents of 5-CQA

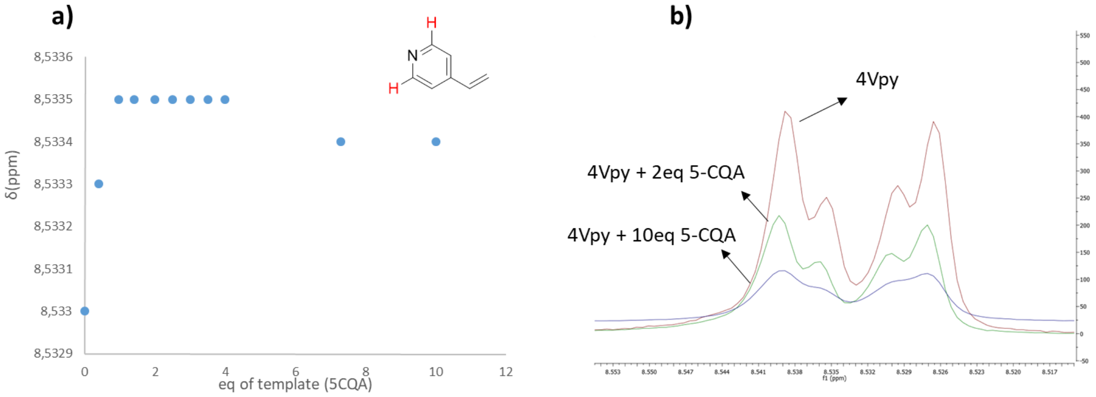

Figure S4. a) Chemical shift variation of aromatic proton of 4-vinypiridine (4Vpy) upon interaction with template molecule (5-CQA). b) progressive shift of the proton aromatic protons adjacent to the nitrogen atom of the pyridine ring in the ^{1}H-NMR spectra. Pure 4VPy (red), addition of 2eq of 5-CQA (green) and 10eq (blue).

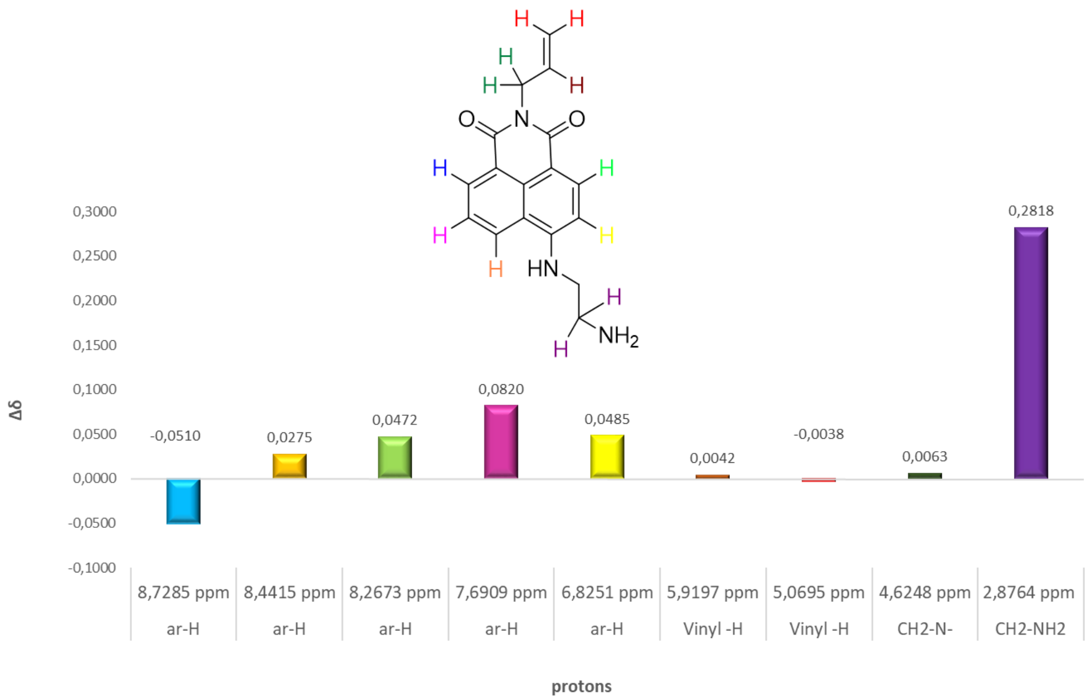

Figure S5. Histogram of the chemical shift variations in the ^{1}H-NMR spectrum of **7** upon progressive additions of 10eq of 5-CQA

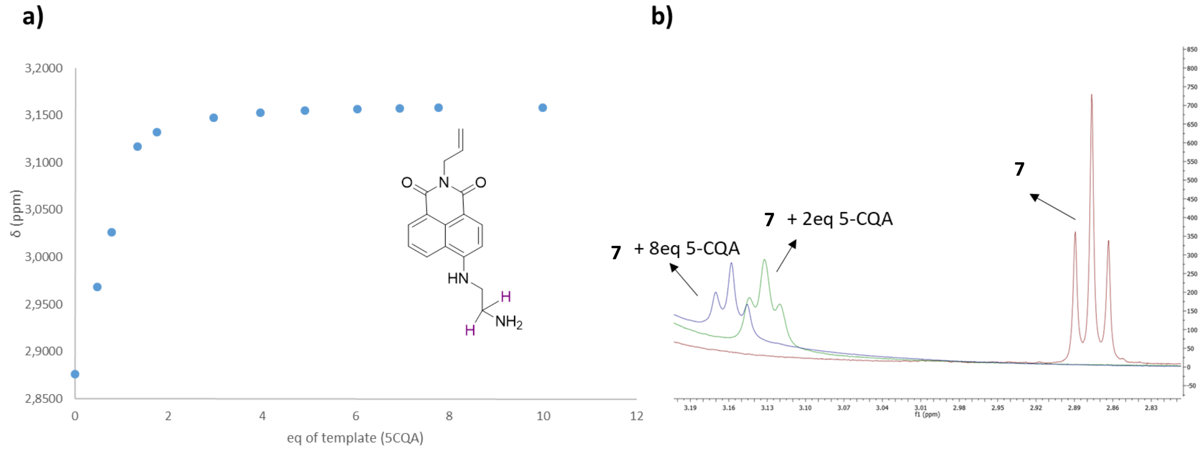

Figure S6. a) Chemical shift variation of CH_2NH_2 protons of monomer **7** upon interaction with 5-CQA; b) progressive shift of protons CH_2NH_2 in the ^{1}H-NMR spectra.

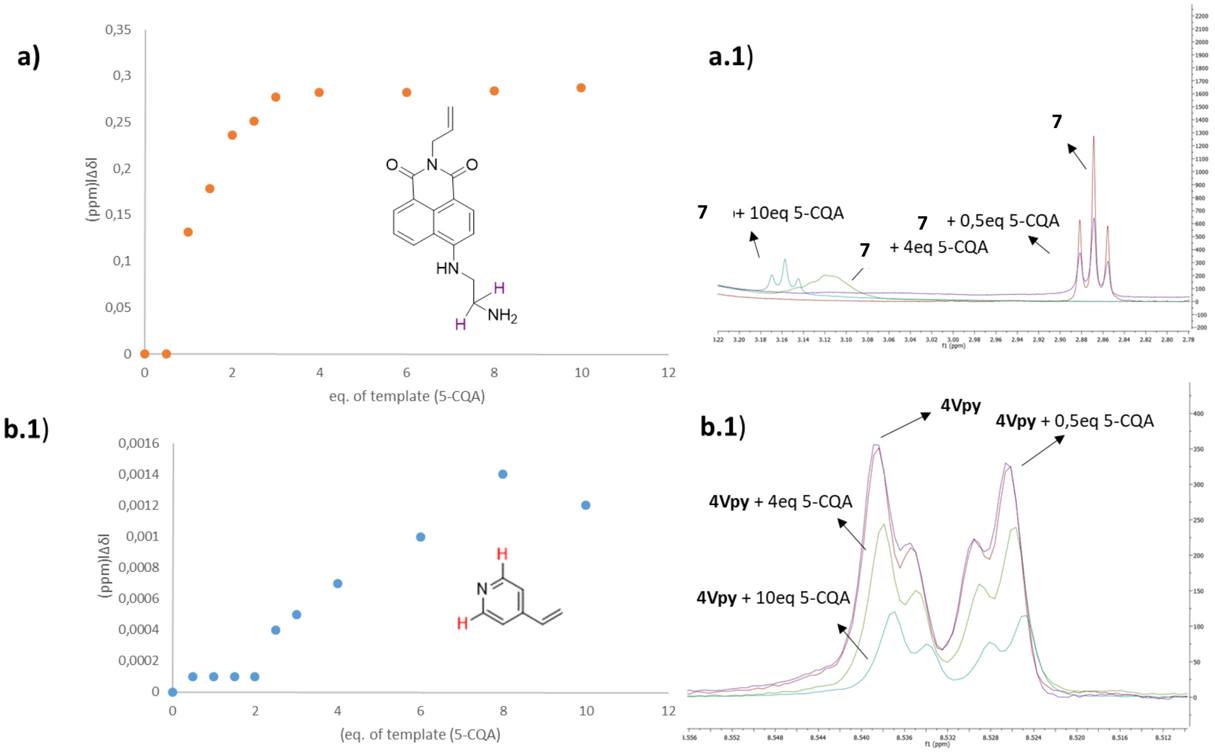

Figure S7: **a)** Chemical shift variation of CH_2NH_2 protons of monomer **7** upon interaction of a monomers mix with template molecule (5-CQA). **a.1)** progressive shift of the protons CH_2NH_2 in the ^{1}H-NMR spectra: **7** (red), addition of 0.5eq of 5-CQA (purple), 4eq (green) and 10q (blue). **b)** Chemical shift variation of aromatic proton of 4VP upon interaction of a mixture of monomers and the template molecule (5-CQA). **b.1)** progressive shift of the proton Ar-**H** in the ^{1}H-NMR spectra. 4VP (red), addition of 0.5eq of 5-CQA (purple), 4eq (green) and 10q (blue).

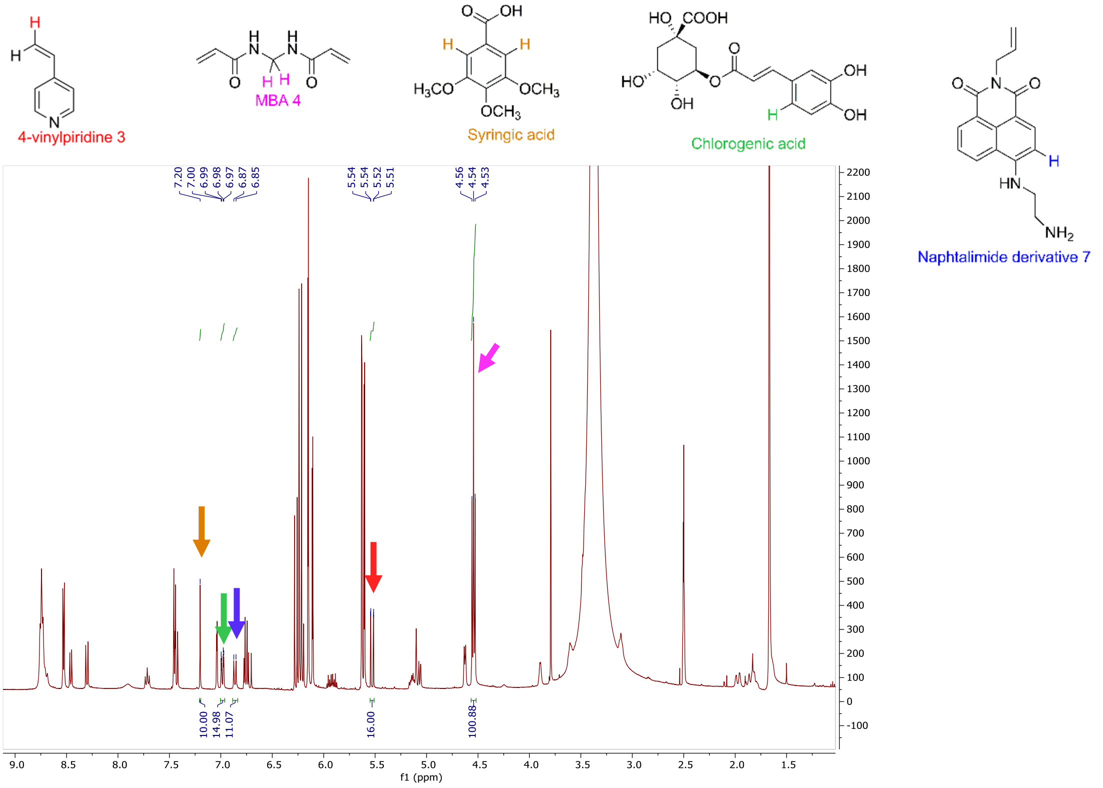

Figure S8: Mixture of monomers 4VP **3** and naphtalimide derivative **7**, cross-linker MBA **4**, syringic acid (internal standard) and 5-CQA in DMSO–d6 before polymerization

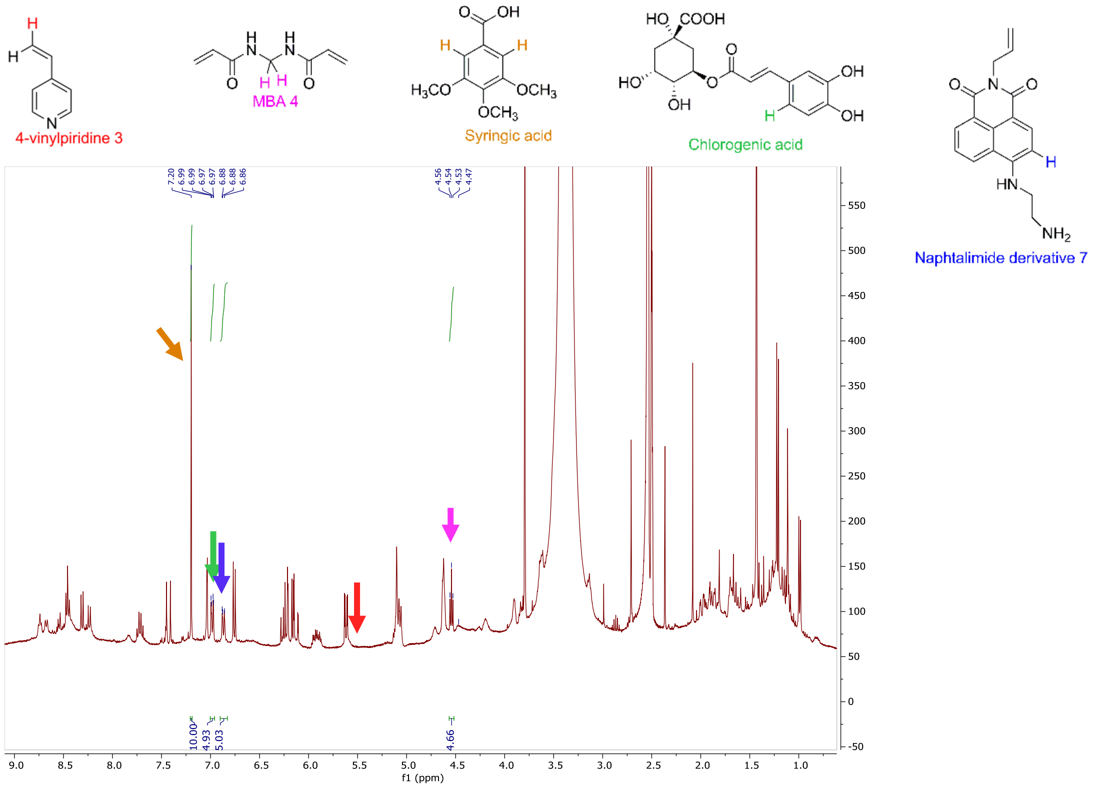

Figure S9: Mixture of monomers 4VP **3** and naphtalimide derivative **7**, cross-linker MBA **4**, syringic acid (internal standard) and 5-CQA in DMSO–d6 after polymerization

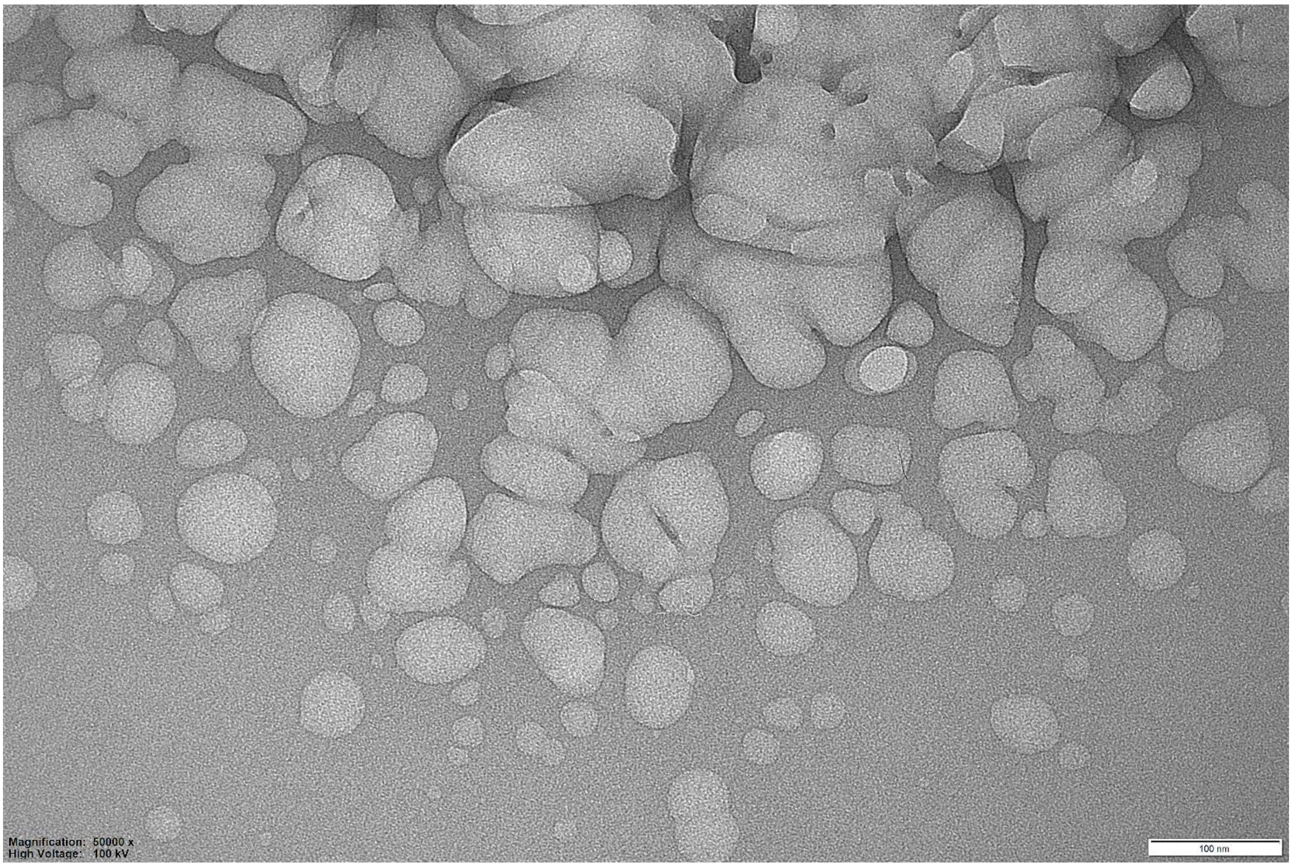

Figure S10: TEM image of fMIP03

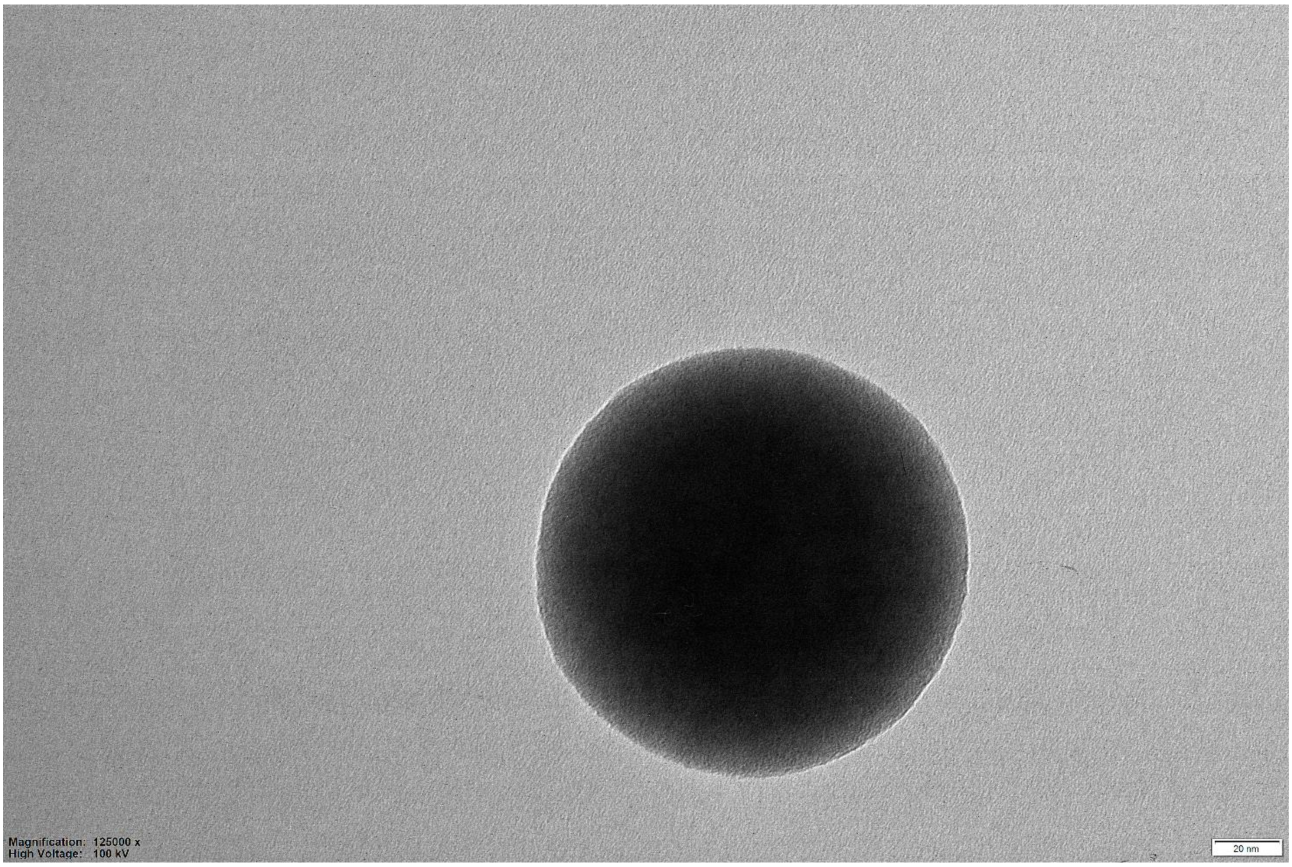

Figure S11: TEM image of fMIP03
